# Topological Deep Learning: A New Dimension in Gastroenterology for Metabolic Dysfunction-Associated Fatty Liver

**DOI:** 10.7759/cureus.60532

**Published:** 2024-05-17

**Authors:** Yashbir Singh, Ranya Ammar, Mostafa Shehata

**Affiliations:** 1 Radiology, Mayo Clinic, Rochester, USA; 2 Pediatric Medicine, New Medical Centre Hospital, Abu Dhabi, ARE; 3 Gastroenterology, Sheikh Shakhbout Medical City, Abu Dhabi, ARE

**Keywords:** computational mathematics, diagnostic imaging, personalized medicine, gastroenterology, medical imaging, metabolic dysfunction associated liver diseases, topological deep learning

## Abstract

Topological deep learning (TDL) introduces a novel approach to enhancing diagnostic and monitoring processes for metabolic dysfunction-associated fatty liver disease (MAFLD), a condition that is increasingly prevalent globally and a leading cause of liver transplantation. This editorial explores the integration of topology, a branch of mathematics focused on spatial properties preserved under continuous transformations, with deep learning models to improve the accuracy and efficacy of MAFLD diagnosis and staging from medical imaging. TDL's ability to recognize complex patterns in imaging data that traditional methods might miss can lead to earlier and more precise detection, personalized treatment, and potentially better patient outcomes. Challenges remain, particularly regarding the computational demands and the interpretability of TDL outputs, which necessitate further research and development for clinical application. The potential of TDL to transform the gastroenterological landscape marks a significant step toward the incorporation of advanced mathematical methodologies in medical practice.

## Editorial

Since June 2023, the term for non-alcoholic fatty liver disease (NAFLD) has been updated to metabolic dysfunction-associated steatotic liver disease (MASLD). MASLD is now the most common liver disease globally, with an estimated prevalence of 32%, and is a primary cause of liver-related morbidity and mortality [1,2]. The prevalence of MASLD varies significantly across different world regions, influenced by obesity rates, genetics, and socioeconomic factors. Recent studies have estimated the incidence rate at 46 per 1000 person-years (95% confidence interval (CI): 39-53), with overweight or obese individuals facing a threefold increased risk of developing MASLD [3]. The high prevalence of heterogeneity and its broad impact on multiple organs underscore the need for precise diagnostic and prognostic tools. Traditional diagnostic methods, including liver biopsy, are invasive and often not ideal for early detection or population-level screening. Conversely, non-invasive markers of fibrosis, along with FibroScan (Echosens SA, Paris, France) and MRI elastography, are valid diagnostic tools. Recent advances in topological deep learning (TDL) offer a new dimension in the non-invasive diagnosis and staging of fatty liver disease, providing a significant improvement over traditional image analysis methods and introducing TDL-based techniques.

Deep learning in medical imaging

Deep learning has revolutionized the field of medical imaging by providing tools that can automatically detect patterns and anomalies that may elude human eyes [4]. However, standard convolutional neural networks primarily capture patterns based on local pixel intensities, which can lead to significant information loss about the overall geometric and topological structure of tissues [5], information that can be critical in understanding complex diseases like MASLD.

Why TDL?

**Figure 1:**
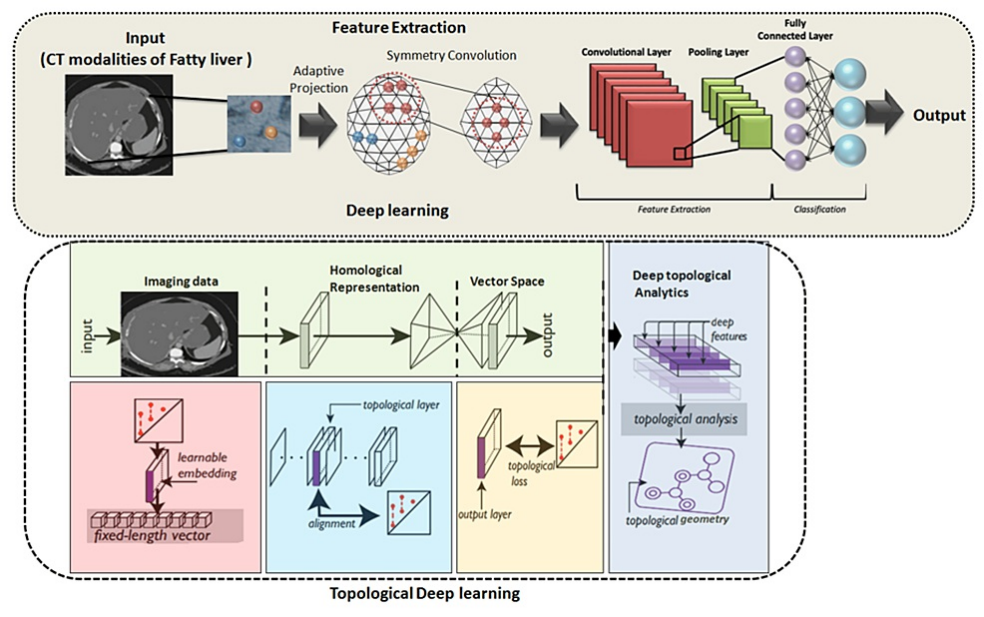
Workflow of deep learning (convolutional neural network) and topological deep learning The workflows of both a conventional convolutional neural network (CNN) and a topological deep learning (TDL) network applied to image analysis of fatty liver. Image Credit: Author Yashbir Singh

TDL integrates the mathematical framework of topology with deep learning techniques (Figure 1) [5,6]. Topology, concerned with the properties of space that are preserved under continuous deformations, adds a powerful lens to analyze medical data [7]. It focuses on features like continuity and holes in data, which are robust against noise and effective descriptors of tissues' structural properties. For fatty liver disease, TDL can enhance the analysis of liver ultrasonography, MRI, or CT images by helping to more accurately characterize and quantify the extent of fat deposition and fibrosis [5,8].

Here's how TDL can impact the management of fatty liver disease

Improved Diagnostic Accuracy

By learning the complex topological structures present in liver images, TDL models can differentiate between healthy and diseased tissues with higher accuracy than traditional models.

Staging and Progression Monitoring

TDL can assist in identifying fatty liver and staging the disease by recognizing topological patterns corresponding to various fibrosis levels, which is crucial for determining appropriate interventions.

Personalized Treatment Protocols

With better staging and diagnosis, clinicians can tailor treatments based on the specific characteristics of the liver’s condition, potentially improving outcomes through personalized medicine.

Non-Invasive Screening

Utilizing TDL in routine screening could facilitate the early detection of MASLD in asymptomatic patients using non-invasive imaging tests rather than biopsies.

Challenges ahead

Despite its promise, incorporating TDL into clinical practice presents challenges. First, the computational complexity of TDL models requires substantial processing power, which can be a barrier in settings with limited resources. Moreover, the interpretability of TDL outputs is often complex due to the abstract nature of topological features. Educating clinicians and integrating insights from TDL models into clinical workflows will require concerted efforts in the training and development of user-friendly interfaces.

Conclusion

TDL represents a transformative approach to diagnosing and managing fatty liver disease. By leveraging the intrinsic geometric structures of liver images, TDL offers a robust framework for improving accuracy in diagnosis, monitoring disease progression, and personalizing treatment plans. However, the success of TDL in clinical settings will depend on overcoming computational and educational barriers and on the development of partnerships between technologists, clinicians, and mathematicians. It is crucial to continue research in this innovative area as we advance, ensuring that technological advancements align with clinical needs and ultimately enhance patient care. This emerging toolset promises to advance gastroenterology and offer new insights into the broader medical diagnostics and therapeutics field, marking a new era in the intersection of computational mathematics and medicine.
